# Molecular Signatures of Early-Onset Bipolar Disorder and Schizophrenia: Transcriptomic and Machine-Learning Insights into Calcium and cAMP Signaling, Including Sex-Specific Patterns

**DOI:** 10.3390/ijms262412109

**Published:** 2025-12-16

**Authors:** Sara Sadat Afjeh, Sohom Dey, Daniel Kiss, Marcos Sanches, Fernanda Dos Santos, Jennie G. Pouget, Niki Akbarian, Shreejoy Tripathy, Vanessa F. Gonçalves, James L. Kennedy

**Affiliations:** 1 Campbell Family Mental Health Research Institute, Centre for Addiction and Mental Health (CAMH), Toronto, ON M5T 1R8, Canadafernanda.santos@camh.ca (F.D.S.); jennie.pouget@mail.utoronto.ca (J.G.P.); vanessa.goncalves@camh.ca (V.F.G.); 2 Krembil Centre for Neuroinformatics, Centre for Addiction and Mental Health (CAMH), Toronto, ON M5T 1R8, Canada; 3 Centre for Addiction and Mental Health (CAMH), Biostatistics Core, Dalla Lana School of Public Health, University of Toronto, Toronto, ON M6J 1H4, Canada; marcos.sanches@camh.ca; 4 Department of Psychiatry, University of Toronto, Toronto, ON M5S 1A1, Canada

**Keywords:** bipolar disorder, schizophrenia, age of onset, transcriptomics, calcium signaling, co-Expression networks, machine learning

## Abstract

Early age of onset is a major predictor of poor disease course in Bipolar Disorder (BD) and Schizophrenia (SCZ), often associated with greater symptom severity, cognitive decline, and worse outcomes. However, the biological mechanisms that shape age- and sex-specific vulnerability remain unclear, limiting progress toward early identification and intervention. To address this gap, we conducted an integrative transcriptomic study of 369 postmortem dorsolateral prefrontal cortex samples from the CommonMind Consortium. Differential gene expression, Weighted Gene Co-Expression Network Analysis, and gene set enrichment analysis were applied to identify pathways associated with age of onset, complemented by sex-stratified models and cellular deconvolution. To assess predictive signals, we applied a rigorous two-stage machine-learning framework using nested cross-validation, with Lasso feature selection followed by L2-regularized logistic classification. Performance was evaluated solely on held-out test folds. Genes and modules linked to earlier onset showed consistent enrichment for calcium signaling, with downregulation of *CACNA1C* and multiple adenylate-cyclase-related transcripts, while female-specific analyses revealed selective dysregulation of cyclase-associated pathways. Network analysis identified a calcium-enriched module associated with onset and sex, and diagnosis-specific modeling highlighted *MAP2K7* in early-onset BD. The predictive model achieved an AUC of 0.63, and the top 50 machine-learning features were significantly enriched in calcium signaling pathway. These findings converge on calcium–cAMP signaling networks as key drivers of early psychiatric vulnerability and suggest biomarkers for precision-targeted interventions.

## 1. Introduction

Bipolar Disorder (BD) and Schizophrenia (SCZ) are severe psychiatric disorders with profound impacts on cognition, affect regulation, and long-term psychosocial functioning. While they exhibit distinct clinical features, both disorders share considerable genetic architecture and molecular overlap, particularly in neurodevelopmental and synaptic pathways [1,2,3].

One of the strongest clinical predictors of illness trajectory in BD and SCZ is age at onset [4,5]. Definitions of early onset in BD vary across studies. Large epidemiological cohorts commonly use an onset before 21 years, whereas investigations emphasizing neurodevelopmental vulnerability or pediatric bipolar disorder apply more conservative thresholds of ≤17 or <18 years, corresponding to the transition from adolescence to early adulthood [6,7,8]. This spectrum captures a developmental continuum, with ≤17 years denoting very-early/pediatric onset and 18–21 years reflecting adolescent/early-adult onset. Empirical evidence supports the clinical validity of the <18-year demarcation, as this subgroup demonstrates greater familial loading, earlier prodromal symptoms, and increased neurodevelopmental burden [6,7,8].

In contrast, schizophrenia definitions are more consistent: early-onset SCZ is typically defined as onset before 18 years, and very-early onset before 13 years [9,10].

Additionally, sex differences in age of onset and symptom profiles are well documented as females tend to exhibit later onset, distinct mood symptomatology, and variable treatment outcomes compared to males [11,12,13,14]. However, the molecular mechanisms mediating these age- and sex-specific differences are incompletely understood [15].

The emergence of high-resolution transcriptomic technologies such as RNA sequencing (RNA-seq) has enabled the interrogation of postmortem brain tissues to uncover the gene expression landscapes underlying psychiatric illness [16]. Among the biological pathways implicated, calcium signaling has consistently emerged as a key regulator of synaptic plasticity, neurotransmission, and neuronal excitability [17,18,19,20]. Multiple large-scale genome-wide association studies (GWAS) have identified variants in calcium channel genes, including *CACNA1C*, as robust risk factors for both BD and SCZ [21,22,23]. Dysregulation of calcium-dependent signaling cascades may therefore contribute to disease onset and progression [18,19,24,25,26].

In parallel, recent studies have begun to elucidate sex-biased gene expression in the brain, with implications for neurodevelopmental timing and psychiatric risk [27,28]. For instance, hormone-responsive signaling pathways, including those involving adenylate cyclases and cAMP signaling, may modulate sex-dependent vulnerability via differential regulation of calcium flux and receptor activation [27,29,30].

To comprehensively understand these relationships, integrative approaches are required. Systems-level methods such as Weighted Gene Co-Expression Network Analysis (WGCNA) allow for the identification of biologically coherent gene modules that correlate with clinical phenotypes such as age of onset [31,32,33]. Additionally, machine learning models offer powerful tools for prioritizing predictive biomarkers from high-dimensional transcriptomic data [34,35].

Here, we present a multi-layered transcriptomic analysis of postmortem dorsolateral prefrontal cortex (DLPFC) samples from the CommonMind Consortium (CMC), building on prior DLPFC transcriptomic studies in schizophrenia and bipolar disorder [16,36,37,38], with the aim of identifying gene expression signatures associated with early-onset BD and SCZ. We incorporate sex-stratified enrichment analysis, co-expression networks, and predictive modeling to uncover calcium- and cyclase-related pathways underlying disease onset. We adjusted for cellular composition to ensure biological validity.

While our primary focus was on identifying convergent pathways underlying calcium dysregulation in early-onset psychiatric illness, prior work has shown that calcium influx can engage cAMP-dependent and MAPK/JNK signaling cascades, including MAP2K7–JNK pathways, thereby linking second-messenger dynamics to synaptic plasticity and neurodevelopmental vulnerability to schizophrenia and related disorders [39,40,41].

Accordingly, the objective of this study was to systematically characterize transcriptomic signatures associated with early-onset bipolar disorder and schizophrenia using postmortem DLPFC RNA-seq data from the CommonMind Consortium. To achieve this, we implemented a multi-layered analytical framework integrating differential gene expression, gene set enrichment analysis (GSEA), WGCNA, and machine-learning–based feature selection. This integrative design enabled us to evaluate whether calcium-related pathways and other convergent molecular mechanisms, emerge consistently across independent analytic layers, thereby providing a comprehensive validation of our hypothesis. In secondary analyses, we also evaluated sex-specific patterns to determine whether early-onset molecular signatures show distinct profiles in males and females.

## 2. Results

### 2.1. Gene Filtering and Expression Distribution

After filtering out lowly expressed genes, the total number of genes was reduced from 57,820 to 15,862, resulting in a more biologically informative subset. Duplicate samples (*n* = 66) from overlapping brain donors were excluded, yielding a final dataset of 369 unique individuals (106 with BD and 263 with SCZ). Demographic and clinical characteristics of the study cohort are summarized in Table 1 (see Supplementary Table S1 for brain bank details).

#### Batch Effect Correction and PCA Analysis

To evaluate and correct for potential batch-related confounding in the RNA-seq data, we applied ComBat-seq and examined batch effects using principal component analysis (PCA). To better visualize the batch-related effects on gene expression, we plotted PC1 vs. PC2 and PC2 vs. PC3 for each batch. Before batch correction, the PCA revealed a clear separation between batches, with distinct clustering of samples based on their contributor sites (NIMH_HBCC, BrainGVEX, and LIBD_szControl) (see Supplementary Figure S1A,B), indicating the presence of significant batch effects in the raw data. After applying ComBat-seq(), PCA was repeated, showing a marked reduction in batch-related variability, with samples clustering more uniformly, which confirmed the successful correction of batch effects (see Supplementary Figure S1C,D). To assess normalization and prepare the data for downstream analysis, the voom transformation was applied (Supplementary Figure S1E).

### 2.2. Differential Gene Expression and Gene Set Enrichment Analysis

To ensure that multicollinearity would not bias our regression estimates, we calculated variance inflation factors (VIFs) for all predictors in the full model. All variables exhibited VIFs well below the commonly accepted threshold of 5, with a maximum value of 1.15, indicating negligible collinearity. These results support the interpretability of the individual regression coefficients in our transcriptomic models.

**Figure 1:**
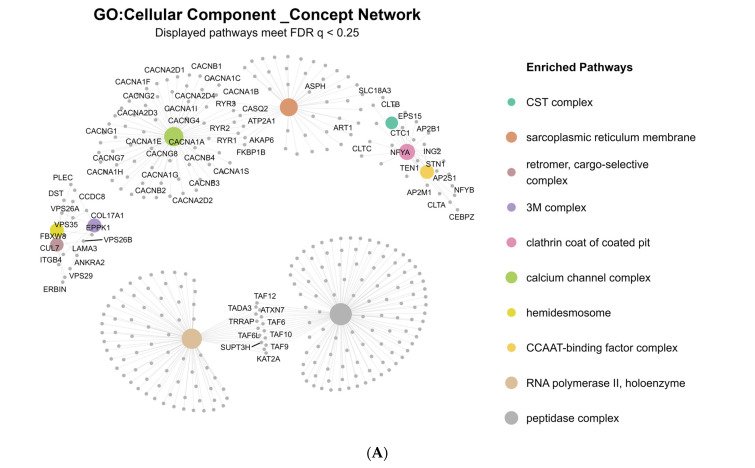

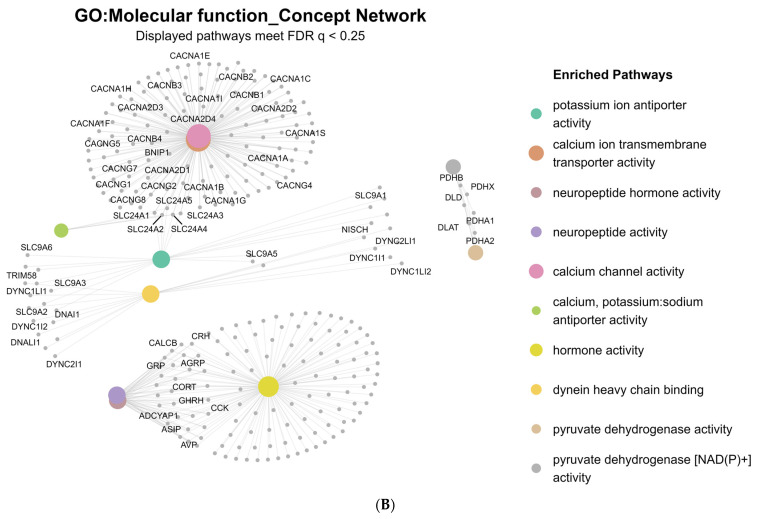
Gene expression pathway enrichment networks generated from the transcriptomic analysis of age of onset in 369 postmortem dorsolateral prefrontal cortex samples. These networks visualize the major biological processes and molecular functions identified through gene set enrichment analysis of genome-wide expression data. In both panels, each colored central node represents one enriched biological pathway, gray nodes represent genes contributing to that pathway, node size corresponds to the number of genes included in the pathway, and connecting lines indicate gene–pathway membership. Only pathways with a false discovery rate–adjusted q value below 0.25 are displayed. (**A**) Cellular Component Enrichment Network. This network represents the leading enriched pathways classified under the Cellular Component domain. For interpretability, each pathway displays its ten most influential contributing genes, with additional biologically important genes (such as members of the CACNA calcium channel family) retained due to their central role in neuronal signaling. The analysis revealed strong enrichment of calcium-related structural components, including the calcium channel complex (normalized enrichment score = −1.85, *p* = 8.46 × 10^−4^) and the voltage-gated calcium channel complex (normalized enrichment score = −1.88, *p* = 2.14 × 10^−3^). (**B**) Molecular Function Enrichment Network. This concept network highlights the most significantly enriched molecular functions. Larger hubs indicate pathways composed of more genes, and the structure of the network emphasizes coordinated dysregulation across related functional themes. Notably, there was strong enrichment in calcium ion transmembrane transporter activity (normalized enrichment score = −1.86, *p* = 1.03 × 10^−4^) and calcium channel activity (normalized enrichment score = −1.87, *p* = 1.23 × 10^−4^), driven largely by extensive involvement of CACNA and CACNB gene families.

Differential gene expression analysis identified multiple pathways related to calcium signaling as significantly dysregulated in association with earlier age of onset in BD and SCZ. GSEA of Cellular Component (CC) terms revealed prominent downregulation of Voltage-gated calcium channel complex (GO:0005891), and Calcium channel complex (GO:0034704) (Figure 1A).

Similarly, GSEA for Molecular Function (MF) terms demonstrated dysregulation of Calcium ion transmembrane transporter activity (GO:0015085), and Calcium channel activity (GO:0005262) (Figure 1B).

### 2.3. Sex-Stratified Transcriptomic Analysis

**Figure 2:**
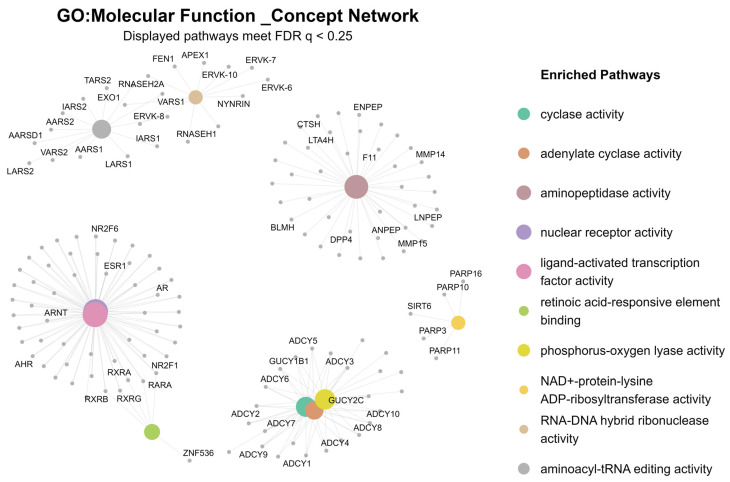
Molecular Function enrichment network (female subset, *n* = 178). This network visualization illustrates the top enriched Gene Ontology (GO) Molecular Function terms identified by GSEA in the female subset. Nodes represent either GO terms (large colored hubs) or their associated genes (gray peripheral nodes), with edges indicating membership relationships. Several molecular functions show strong positive enrichment, including cyclase activity (NES = 2.06, *p* = 1.14 × 10^−5^) and adenylate cyclase activity (NES = 2.03, *p* = 1.64 × 10^−5^), highlighting coordinated upregulation of ADCY family members.

Sex-stratified analyses revealed significant molecular differences specific to female subset (*n* = 178). GSEA results for this group showed notable enrichment for calcium- and cyclase-related molecular functions, with enrichment observed for cyclase activity (GO:0009975) and adenylate cyclase activity (GO:0004016) (Figure 2).

### 2.4. Weighted Gene Co-Expression Network Analysis (WGCNA)

**Figure 3:**
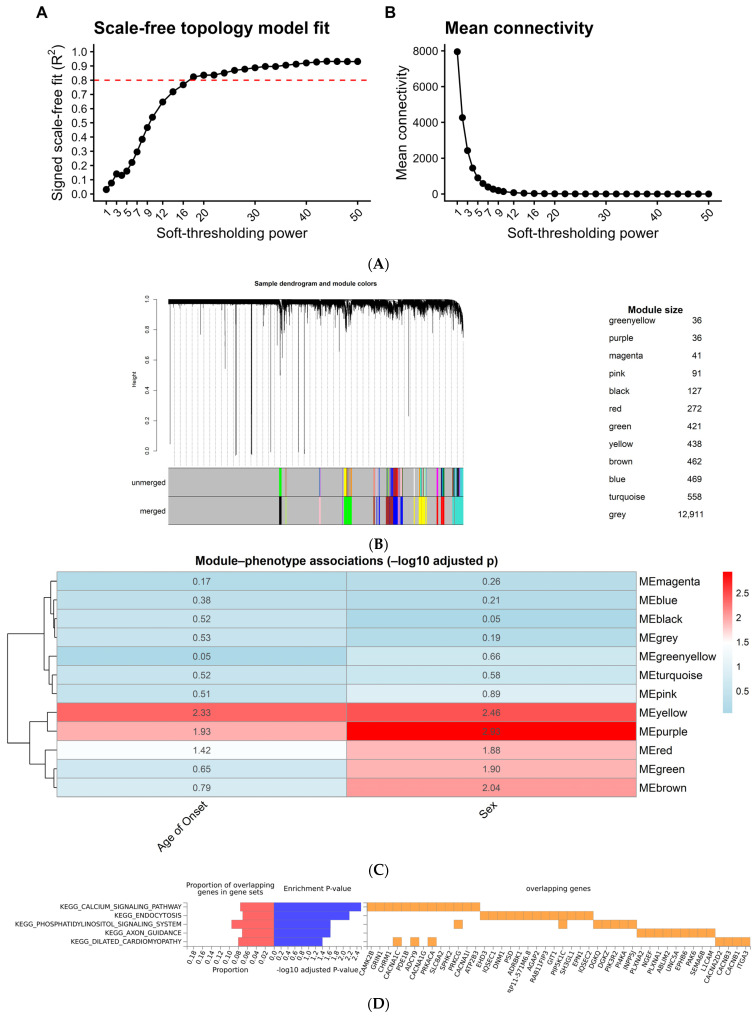
(**A**) Scale-free topology model fit plot displaying soft-thresholding power on the *X*-axis and signed R^2^ on the *Y*-axis. Dots represent different soft power values tested for constructing the weighted gene co-expression network. The red line represents the commonly used threshold of R^2^ ≥ 0.80, beyond which the network approximates a scale-free topology. A soft power of 18 was selected for the full dataset of BD and SCZ samples (*n* = 369), as it is the lowest value where the scale-free topology criterion is met. (A-Down) Mean connectivity plot displaying soft-thresholding power on the *X*-axis and mean network connectivity on the *Y*-axis. Dots represent the mean connectivity at each power value. As soft power increases, mean connectivity declines, indicating a reduction in overall network density. This trend reflects the expected effect of raising the soft threshold, ensuring that highly connected genes retain meaningful biological relationships while reducing spurious connections. (**B**) Cluster dendrogram displaying hierarchical clustering of genes based on co-expression patterns. The vertical axis represents the dissimilarity (1—correlation) between genes, while the branches indicate clusters of highly co-expressed genes. Initial unmerged modules are shown in the lower gray panel, while the merged modules (following dynamic tree cut and module merging) are displayed in the colored panel beneath. Module composition table showing the number of genes assigned to each co-expression module. A total of 12 co-expressed gene modules were identified, each represented by a distinct color. The grey module contains unassigned genes that did not cluster into any specific module. (**C**) Heatmap of Adjusted *p*-values for Module–Trait Associations. The heatmap displays the adjusted *p*-values (log10 scale) for module–trait correlations in the full dataset (*n* = 369 BD and SCZ samples). The color gradient represents the level of statistical significance, with blue indicating higher *p*-values (weaker associations) and red indicating lower *p*-values (stronger associations). Notably two modules (purple, and yellow) exhibited significant correlations with age of onset. (**D**) Functional Enrichment Analysis of Genes in the Yellow Module (*n* = 369) Using FUMA. The plot presents the KEGG pathway enrichment results for genes included in the yellow module of the SCZ and dataset (*n* = 369). The yellow module was significantly enriched in calcium signaling pathways.

WGCNA was performed to identify gene modules associated with age of onset and sex. Soft-thresholding analysis confirmed a scale-free network topology, with the optimal soft-thresholding power selected at 18, achieving an R^2^ > 0.8 (Figure 3A).

Hierarchical clustering and dynamic tree cutting identified 12 distinct co-expression modules after merging similar modules (Figure 3B). Module-trait correlation analysis revealed significant associations between specific modules and clinical traits. The purple module and the yellow module showed significant correlation with both age of onset and sex (Figure 3C). Enrichment analysis of yellow module genes (via FUMA) confirmed enrichment in KEGG_Calcium_Signaling_Pathway, including *CACNA1C* gene (module–trait correlation *p* = 0.003) (Figure 3D).

### 2.5. WGCNA for Bipolar Disorder Samples

When the WGCNA’s analysis was limited to the BD samples (*n* = 106), 11 distinct co-expression modules were identified, with the yellow and purple modules showing the significant correlation with age of onset (see Supplementary Figure S2A). Notably, the yellow module was again enriched in calcium signaling pathways, containing key genes such as *ATP2B2*, *CAMK2A*, *CACNA1B*, *GRIN1*, and *ADCY1.* The purple module did not show enrichment for any specific biological pathways (see Supplementary Figure S2B).

### 2.6. Cell Type Proportion Analysis

To ensure that our findings accurately reflect the biological processes related to the studied phenotypes, we estimated rCTPs from the bulk RNA-seq data, and compared them with gene expression patterns. This comparison confirmed that the significant findings in our gene expression analysis were not confounded by variations in cellular composition.

### 2.7. Predicting Early-Onset Schizophrenia and Bipolar Disorder Using Machine Learning for Transcriptomic Feature Selection

In this study, we applied a two-stage machine-learning framework to identify transcriptomic features associated with early-onset schizophrenia and bipolar disorder and to evaluate their predictive value. The dataset included 369 individuals, and the workflow consisted of (1) feature selection using LASSO regression applied to the continuous age-at-onset variable and (2) classification of early-onset versus late-onset illness using Logistic Regression with L2 regularization. Feature selection and model tuning were performed within the inner loop of a nested cross-validation design, while model evaluation was conducted on the independent outer test folds. This nested setup ensured strict separation between training, tuning, and testing to prevent information leakage and to obtain unbiased performance estimates. Further details in the methods section.

**Figure 4:**
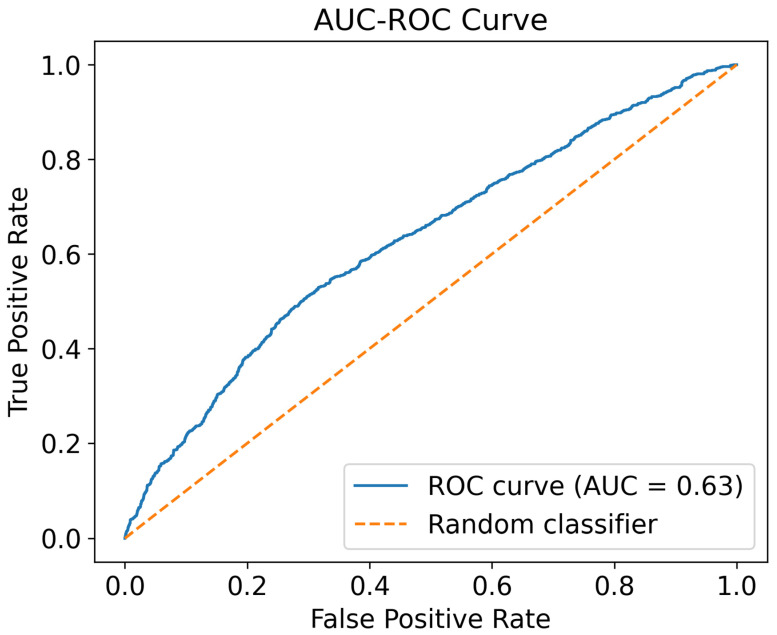
Receiver Operating Characteristic (ROC) curve for the LASSO-based predictive model of early- vs. late-onset cases. This figure presents the Receiver Operating Characteristic (ROC) curve obtained from the outer test folds of the repeated nested cross-validation framework applied to transcriptomic data from 369 individuals with schizophrenia or bipolar disorder. The two-stage modeling pipeline consisted of (1) LASSO regression for feature selection using the continuous age-at-onset variable and (2) Logistic Regression with L2 regularization for classifying early-onset (<18 years) versus late-onset illness. All hyperparameters for both stages were tuned exclusively within the inner loop to prevent information leakage, while performance was evaluated on the independent outer test folds. The model achieved a mean AUC of 0.63 across all outer folds and repetitions, indicating moderate discrimination between early- and late-onset cases based on transcriptomic profiles. The blue curve represents the aggregated ROC performance of the model, while the orange dashed diagonal denotes chance-level classification (AUC = 0.50).

Using this framework, the model achieved a mean AUC of 0.63, reflecting moderate discrimination between early- and late-onset cases based on transcriptomic profiles (Figure 4). Stability analysis of the LASSO-selected features revealed that two genes were consistently selected in all 50 outer test folds of the repeated nested cross-validation procedure, demonstrating exceptionally high robustness to data partitioning and hyperparameter variation. An additional 17 genes were selected in at least 50% of outer folds, indicating a broader set of moderately stable transcriptomic predictors. Enrichment analysis of the top 50 most frequently selected features further revealed significant overrepresentation of calcium signaling pathways, highlighting the biological coherence of the model’s predictive markers (see Supplementary Table S2).

These results show that the two-stage modeling strategy, combined with repeated nested cross-validation, identified a small set of highly stable features and a larger set of moderately stable features while achieving reliable predictive performance.

### 2.8. Interaction Model: Age of Onset × Primary Diagnosis Uncovers Disorder-Specific Transcriptional Signatures

**Figure 5:**
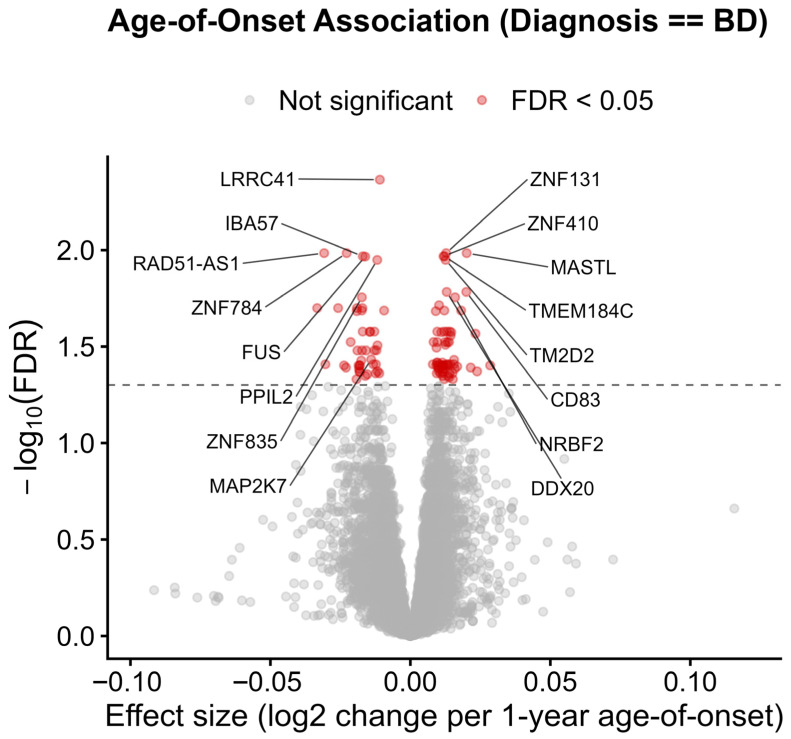
Volcano plot showing gene-expression associations with age of onset in Bipolar Disorder, derived from an interaction model (Age of Onset × Diagnosis) applied to CMC DLPFC tissue (*n* = 369; total genes tested = 15,862). The x-axis reflects the BD-specific slope (log2 fold-change in expression per 1-year difference in onset age), and the y-axis displays –log10(FDR). Covariates included sex, race, PMI, pH, RIN, and age at death. A total of 112 genes surpassed the FDR < 0.05 threshold (red), including 68 up-regulated and 44 down-regulated genes. Significant hits include *MAP2K7*, a Ca^2+^-responsive MAPK/JNK kinase, and calcium-linked stress regulators such as *IBA57*, *FUS*, and *NRBF2*. Additional significant genes (e.g., *TMEM184C*, *DDX20*, *CD83*) participate in GPCR/cAMP-related signaling. Together, the findings highlight dysregulation of calcium-modulated and MAPK-associated pathways in earlier age-of-onset BD.

To interrogate diagnosis-specific molecular trajectories linked to illness onset, we incorporated an Age-of-Onset × Primary Diagnosis interaction term into the linear model. This framework enabled us to test whether the association between gene expression and age of onset differs between BD and SCZ, thereby capturing disorder-specific transcriptional responses to early onset. The interaction model identified 112 genes with significant diagnosis-by-onset effects (FDR < 0.05), indicating that these transcripts exhibit divergent molecular slopes in BD versus SCZ as onset occurs earlier (Figure 5).

## 3. Discussion

This study integrates transcriptomic, co-expression, and machine learning methodologies to uncover molecular signatures associated with age of onset in Bipolar Disorder (BD) and Schizophrenia (SCZ), with a central focus on calcium signaling and sex-specific biological mechanisms. Utilizing postmortem dorsolateral prefrontal cortex (DLPFC) RNA-seq data from the CommonMind Consortium (*n* = 369; 106 BD and 263 SCZ), we examined gene expression patterns and their relationship to early disease onset, stratified by sex and diagnosis.

The age of onset across the cohort ranged from 7 to 48 years (mean = 22), and age at death ranged from 17 to 83.4 years (mean = 46). Although this range reflects the characteristics of our postmortem cohort, the pattern of earlier onset being biologically distinct is consistent with the genome-wide association study by Mullins et al. (2021), which reported stronger polygenic contributions and enrichment of calcium-channel–related pathways among individuals with earlier illness onset [42]. Similarly, our transcriptomic analyses identified reduced expression of calcium signaling genes in individuals with earlier onset, supporting convergent evidence across genomic and cortical transcriptomic levels.

Among the 178 female individuals (52 BD, 126 SCZ), the mean ages of onset and death were 22.0 and 45.5 years, respectively. Our integrative framework, incorporating WGCNA and predictive models, prioritized gene modules and molecular features associated with early-onset illness, revealing convergent signatures related to calcium homeostasis and female-specific transcriptomic alterations [1,42,43].

### 3.1. Calcium Channel Dysregulation and Psychiatric Onset

Since the earliest GWAS efforts in psychiatric genetics, convergent evidence has consistently implicated calcium signaling genes as central components of the shared genetic architecture between BD and SCZ. Early landmark GWASs identified *CACNA1C* and other voltage-gated calcium-channel subunits as genome-wide significant loci for BD [44,45], while subsequent large-scale meta-analyses extended these findings to SCZ and cross-disorder cohorts, revealing convergent enrichment of calcium-related pathways [42,43,44,45]. Collectively, these studies have repeatedly implicated *CACNA1C*, *CACNB2*, and *CACNB4* as central regulators of neuronal excitability, synaptic transmission, and calcium-dependent signaling cascades. Integrative transcriptome-wide association (TWAS), eQTL, and pathway-level analyses have further demonstrated that calcium-ion transport, voltage-gated channel activity, and downstream CREB-mediated transcription constitute reproducible biological signatures that transcend diagnostic boundaries, reinforcing calcium signaling as a convergent molecular substrate of major psychiatric illness [45,46]. Our transcriptomic and network findings build upon this well-established genomic foundation, demonstrating that calcium signaling abnormalities are not only genetically encoded but also transcriptionally manifested in postmortem brain tissue of affected individuals. Our study supports and extends accumulating evidence that calcium signaling pathways play a central role in the pathophysiology of early-onset psychiatric disorders. Transcriptomic analyses revealed downregulation of genes involved in calcium ion transport, including *CACNA1C*, *ADCY1*, and *ATP2A3*, particularly in individuals with earlier onset.

Gene Set Enrichment Analysis (GSEA) revealed significant downregulation of calcium signaling pathways. Terms related to the voltage-gated calcium channel complex and calcium ion transmembrane transporter activity were especially enriched. *CACNA1C*, a key voltage-gated calcium channel gene encoding the Cav1.2 subunit, showed reduced expression associated with early onset. Variants in *CACNA1C* (e.g., rs1006737, rs4765905) have been widely replicated across GWAS, with known associations to altered cortical connectivity, executive dysfunction, and treatment nonresponse in BD and SCZ [26,44]. A recent meta-analysis reaffirmed *CACNA1C* as one of the most consistently replicated risk loci across neuropsychiatric GWAS, with pleiotropic effects on cognition, emotional regulation, and age of onset [26].

Calcium signaling plays a fundamental role in regulating neurodevelopmental trajectories, synaptogenesis, and neurotransmitter release. Disruption of calcium channel activity impairs excitability, delays neuronal differentiation, reduces dendritic complexity, and disrupts the development of inhibitory interneurons, mechanisms central to the pathophysiology of early-onset SCZ and BD [47]. Clinical imaging studies have shown that individuals with early-onset illness exhibit disrupted connectivity and cortical thinning in calcium-sensitive brain regions, including the prefrontal cortex and anterior cingulate cortex [48,49].

We also observed reduced expression of *ATP2A3*, which encodes a SERCA calcium pump responsible for maintaining endoplasmic-reticulum calcium stores and regulating intracellular calcium homeostasis. *ATP2A3* activity contributes to synaptic signaling stability and protection against excitotoxic stress through its role in calcium buffering. Prior studies have shown that dysregulation of ER calcium handling can increase vulnerability to neuroinflammation and cellular stress responses [50]. Our findings suggest that reduced *ATP2A3* expression may reflect altered calcium homeostasis in individuals with earlier onset.

*ADCY1* encodes adenylate cyclase 1, a key enzyme in converting ATP into cyclic AMP (cAMP). This process activates cAMP-dependent signaling cascades involving CREB, a transcription factor central to memory formation, stress adaptation, and emotional regulation [51,52]. 

### 3.2. Calcium- and cAMP-Mediated Molecular Mechanisms in Early-Onset Illness

Together, these findings are consistent with prior evidence suggesting that alterations in calcium-mediated signaling can influence synaptic maturation, neuronal excitability, and neuroplasticity in the prefrontal cortex—processes that have been implicated in the pathophysiology of bipolar disorder and schizophrenia [1,42,43]. In our cohort, earlier illness onset was associated with reduced expression of calcium- and cAMP-related genes in the DLPFC, including *ATP2A3* and *ADCY1.* These genes contribute to intracellular calcium homeostasis and calcium-dependent cAMP–CREB signaling, respectively-pathways with well-established roles in synaptic plasticity and cellular stress responses [50,51,52]. The coordinated reduction of these transcripts in early-onset cases suggests potential convergence on calcium- and cAMP-mediated regulatory systems in cortical circuits, although these observations do not imply direct causality. Rather, they align with existing molecular frameworks linking calcium signaling dysregulation to neurodevelopmental vulnerability in BD and SCZ [40,47,52].

### 3.3. Sex-Specific Transcriptomic Signatures: cAMP and Adenylate Cyclase Activity

Sex-stratified analyses revealed female-specific upregulation of adenylate cyclase genes (*ADCY* genes). These genes regulate cAMP production in response to calcium flux and are central to CREB-mediated transcription. The findings align with both animal and human studies linking female-specific cAMP signaling to psychiatric vulnerability during adolescence, highlighting a potential mechanism for earlier onset and heightened stress reactivity in females [50,51,52].

### 3.4. Co-Expression Network Analysis: Systems-Level Evidence of Calcium Dysregulation

Weighted Gene Co-Expression Network Analysis (WGCNA) revealed 12 distinct gene modules in the full cohort, with the yellow module showing a strong negative correlation with age of onset and sex. This module was enriched in the KEGG Calcium Signaling Pathway and included *CACNA1C* and *ADCY1*, among others. In a BD-specific network (*n* = 106), the same calcium-enriched yellow module was observed, containing key regulators such as *ATP2B2*, *CAMK2A*, *CACNA1B*, and *GRIN1*.

These systems-level findings highlight that calcium pathway disruption is not only a gene-level feature but also embedded in broader co-regulated networks that contribute to early-onset vulnerability. The BD-specific network complements previous SCZ-focused transcriptomic studies such as Gandal et al. (2018), helping to clarify the molecular architecture of early-onset BD, a relatively understudied domain [16].

### 3.5. Predictive Modeling Reveals Key Biomarkers

Our two-stage machine learning model achieved an AUC of 0.63, almost consistent with prior studies demonstrating that values in the 0.65–0.75 range reflect clinically meaningful classification in psychiatric research. For instance, Koutsouleris et al. (2009) demonstrated that neuroanatomical pattern classification could identify individuals at high risk for psychosis with AUCs in this range [53]. Similarly, Antonucci et al. (2022) reported comparable AUCs when using machine learning models to predict functional outcomes in psychiatric populations [54]. These findings underscore the potential utility of gene expression data, even from postmortem tissue, in predicting the neurodevelopmental timing of illness onset. Among the most stable LASSO selected features were *PCDHGA5*, *PCDHGA6*, and *PCDHGA9*, members of the clustered gamma-protocadherin family that encode calcium dependent synaptic adhesion molecules essential for dendritic self-avoidance, synapse organization, and circuit specificity [55,56]. Clustered protocadherins play key roles in establishing neuronal wiring patterns, and their disruption has been linked to altered cortical connectivity and increased susceptibility to neurodevelopmental psychiatric conditions, including BD and SCZ [57,58,59]. The calcium-regulated nature of these genes aligns closely with our broader findings of calcium pathway dysregulation in early-onset illness.

Importantly, our machine-learning analysis converged with the transcriptomic and co-expression results. The top 50 predictive features were significantly enriched for calcium-signaling pathways in KEGG and DAVID analyses [60,61]. This convergence across analytic modalities reinforces calcium-mediated processes as a central molecular axis underlying early-onset vulnerability.

### 3.6. Disorder-Specific Transcriptomic Signatures in Bipolar Disorder

Given the smaller number of BD samples in our dataset and the recognized diagnostic heterogeneity, we conducted a secondary, BD-specific analysis to investigate age-of-onset effects in this group alone. Prior studies indicate that early-onset BD is associated with increased genetic loading, more severe clinical trajectories, higher comorbidity rates, and reduced treatment response. For instance, Kalman et al. (2019) found that early-onset BD individuals exhibit significantly higher polygenic risk scores, suggesting a more heritable, biologically driven subtype [62].

Our findings add transcriptomic support to these observations, showing that early-onset BD is associated with downregulation of calcium signaling components, including *CACNA1C* and *CACNB2*, which are among the most consistently replicated BD risk genes. These disorder-specific results provide a molecular explanation for the clinical observation that early-onset BD often presents with greater mood instability and treatment refractoriness, potentially linked to dysregulated intracellular signaling and altered synaptic plasticity.

Importantly, while our primary WGCNA included both BD and SCZ samples to identify transdiagnostic modules, a separate BD-specific co-expression network was constructed to detect patterns that may be obscured by sample imbalance or diagnostic heterogeneity. This complements prior SCZ-focused network studies and extends the investigation of early-onset molecular signatures to BD, a relatively underexplored area in transcriptomic psychiatry [16].

The interaction model (Age of Onset × Primary Diagnosis) revealed *MAP2K7* as a gene with significant diagnostic specificity, particularly associated with early-onset BD. *MAP2K7* encodes a stress-activated kinase that links calcium influx to JNK/MAPK pathways and modulates synaptic plasticity, oxidative stress responses, and behavioral adaptation [39,40,41,63,64].

This finding suggests that calcium dysregulation in BD may propagate through cAMP and MAPK cascades, forming an extended signaling axis that governs both stress reactivity and neuroplasticity. Additional genes highlighted by this model further support dysregulation in calcium transport, membrane excitability, and synaptic signaling [39,40,41,64].

### 3.7. External Replication in Independent GEO Bipolar Disorder Cohorts

To evaluate the generalizability of our transcriptomic findings, we performed an external validation analysis using two independent microarray datasets from the Gene Expression Omnibus (GSE5388 and GSE5389). These datasets contain a combined total of 40 postmortem BD samples profiled using Affymetrix HG-U133A microarrays [65]. Although phenotypic information was not available in structured form, we manually extracted age of onset, sex, age at death, and postmortem interval from sample metadata.

Despite the small sample size and older microarray platform, the GEO dataset showed convergent signals with our CMC RNA-seq findings. Differential expression analysis using array-weighting (AW) did not yield FDR-significant features, but the two top uncorrected associations (*CAP1* and *AK4*; *p* < 1 × 10^−5^) which map to the cyclase and cAMP signaling axis [66,67], consistent with our transcriptomic findings (Supplementary Table S3). Gene set enrichment analysis further implicated calcium-related pathways, including endoplasmic reticulum calcium ion homeostasis, regulation of calcium ion transport, adenylate cyclase-activating GPCR signaling, sarcoplasmic reticulum Ca^2+^ release, and cGMP-PKG signaling (Supplementary Figures S3–S6, Supplementary Tables S4–S7).

To strengthen inference, we performed formal meta-analysis combining the CMC BD RNA-seq results with GEO microarray statistics using both Fisher’s combined probability test and a signed, sample-size–weighted Stouffer Z-method. We identified a robust set of 53 high-confidence genes that met all criteria:

(i) FDR < 0.05 in Fisher’s test;
(ii) FDR < 0.05 in signed Stouffer’s test;
(iii) concordant direction of effect in both datasets (Supplementary Figure S7).

These reproducible genes prominently feature calcium-signaling components such as *PCDHGA* family genes, *CAB39*, *PVALB*, *RCAN1*, *RANBP9*, and *NTRK3*, several of which are directly involved in Ca^2+^ buffering, Ca^2+^-dependent phosphatase signaling, Ca^2+^-dependent adhesion, or PLC-γ-mediated Ca^2+^ release [68,69,70,71,72]. These findings provide strong external support for the calcium-signaling dysregulation hypothesis identified in our primary analyses.

Full meta-analytic gene lists are provided in Supplementary Tables S8–S10.

### 3.8. Limitations

Despite the strength of integrating transcriptomic, network, and machine-learning approaches, several limitations warrant consideration. First, detailed clinical information relevant to age-of-onset was not uniformly available across cohorts, limiting the ability to achieve full cross-dataset harmonization and restricting direct replication of some disorder-specific findings. Second, the external bipolar cohorts available for validation were relatively small microarray-based datasets, which reduced statistical power and constrained the extent of phenotypic alignment. Although our conservative cross-cohort analyses yielded a biologically coherent and directionally consistent set of replicated signals, particularly within calcium-related pathways, these results should be interpreted in light of platform differences, batch effects, and incomplete clinical covariates. Finally, while convergence across multiple analytical frameworks increases confidence in the robustness of the findings, future replication in larger, deeply phenotyped, multi-omic cohorts will be essential for establishing generalizability and distinguishing diagnosis-specific from shared molecular signatures.

## 4. Materials and Methods

### 4.1. Dataset

We utilized transcriptomic data from 369 postmortem DLPFC samples from the CMC, comprising 106 BD and 263 SCZ samples. All individuals had complete demographic and clinical data, including age of onset, sex, age of death, race, postmortem interval (PMI), Brain pH, and RNA integrity number (RIN) [73,74,75,76]. Demographic and clinical characteristics of the study cohort are summarized in Table Data were derived from three cohorts: NIMH_HBCC, BrainGVEX, and LIBD_szControl sequenced using the HiSeq2000 platform (see Supplementary Table S1).

Tissue preparation and RNA sequencing methodologies varied across the different studies and cohorts. Comprehensive details regarding the collection, management, and RNA sequencing of postmortem DLPFC samples have been extensively documented in previous publications [16,36,37,38].

### 4.2. Data Processing and Normalization

Raw RNA-seq counts were filtered to retain genes with counts per million (CPM) > 1 in at least 50% of samples using the edgeR v4.2.2 (Bioconductor; accessed on 26 November 2025). All analyses were performed in R v4.4.1 (2024-06-14). Samples with RIN < 6 were excluded. Batch effects were corrected using ComBat-seq (sva package v3.52.0 (Bioconductor; accessed on 26 November 2025)). Duplicate samples (*n* = 66) from overlapping brain donors were removed, and PCA was used to assess correction effectiveness. Normalization and modeling steps incorporated limma package v3.60.4.

Prior to model fitting, we assessed multicollinearity among covariates using variance inflation factor (VIF) analysis. All VIF values were below the commonly accepted threshold of 5, indicating that collinearity was not a concern and that each covariate could be reliably interpreted in the model [76].

A unified linear model was used to examine the association between gene expression and age of onset, while adjusting for relevant demographic and technical covariates. The design matrix included the following predictors:

- Primary Model (Full Cohort):

Design age of onset = β0 + β1 (Age of Onset) + β2 (Primary Diagnosis) + β3 (Age at Death) + β4 (Sex) + β5 (Race) + β6 (PMI) + β7 (pH) + β8 (RIN)

Where:

- β_0_: Intercept term;
- β_1_: Effect of age of onset (continuous variable);
- β_2_: Effect of primary diagnosis (categorical: BD vs. SCZ);
- β3–β_9_: Effects of covariates: age at death, sex, race, postmortem interval (PMI), brain pH, and RNA integrity number (RIN).

To explore sex-specific molecular patterns, we performed a sex-stratified analysis using the same modeling framework separately within male and female subsets. This allowed us to identify genes associated with age of onset within each sex and to investigate potential biological pathways underlying sex-dependent transcriptional signatures. In addition, we extended the model to include a primary diagnosis × age of onset interaction term to detect diagnosis-specific transcriptomic effects.

- Interaction Model (Full Cohort):

Design onset = β0 + β1 (Age of Onset) + β2 (Primary Diagnosis) + β3 (Age of Onset × Primary Diagnosis) + β4 (Age at Death) + β5 (Sex) + β6 (Race) + β7 (PMI) + β8 (pH) + β9 (RIN)

Where:

- β_0_: Intercept term;
- β_1_: Effect of age of onset (continuous variable);
- β_2_: Effect of primary diagnosis (categorical: BD vs. SCZ);
- β_3_: Interaction term: how the relationship between age of onset and gene expression differs by diagnosis;
- β_4_–β_9_: Effects of covariates: age at death, sex, race, postmortem interval (PMI), brain pH, and RNA integrity number (RIN).

#### 4.2.1. Weighted Gene Co-Expression Network Analysis (WGCNA)

To investigate the relationships between co-expressed genes and key clinical traits, such as age of onset and sex, WGCNA was employed [77]. WGCNA constructs co-expression networks by grouping genes based on their expression profiles, identifying modules of highly correlated genes that may share functional similarities [78]. Given the smaller sample size of individuals with BD (*n* = 106) and our focus on disorder-specific molecular architecture, a separate WGCNA was conducted using only BD samples to construct co-expression networks reflective of this diagnostic group.

##### Data Preprocessing and Gene Filtering

To ensure robust data quality, raw RNA-seq counts were first normalized to CPM and processed using the voom transformation to stabilize the mean-variance relationship [79,80]. The GoodSamplesGenes function from the WGCNA R package v1.73 (Bioconductor; accessed on 26 November 2025) [77] was employed to identify and remove any low-quality samples or genes that could negatively impact the downstream analyses [81,82].

##### Network Construction

We used the pickSoftThreshold function to choose a soft-thresholding power (β) that converts gene–gene correlations into network adjacencies by raising |correlation| to the βth power, thereby preserving the graded strength of connections and approximating a scale-free topology [78,81]. The criterion was an R^2^ ≥ 0.80 for scale-free fit with minimal loss of mean connectivity. This procedure yielded β = 18 for the full dataset (*n* = 369) and β = 16 when restricted to the BD subset (*n* = 106).

##### Topological Overlap Matrix (TOM) Transformation

The adjacency matrix was subsequently transformed into a Topological Overlap Matrix (TOM), which provides a robust measure of gene connectivity by considering not only direct connections between genes but also shared neighbors [78,81]. The dissimilarity (1-TOM) was then calculated, which allowed for more accurate clustering of genes into modules. The TOM-based dissimilarity measure was subjected to average linkage hierarchical clustering to identify modules of co-expressed genes, with a minimum module size of 30 genes. Modules with similar expression patterns (based on a dissimilarity threshold of less than 0.25) were merged to reduce redundancy and improve biological interpretability.

##### Module Detection

In the full dataset of 369 BD and SCZ samples, the blockwiseModules() function was used to identify 12 distinct co-expression modules. These modules represent clusters of genes that are highly co-expressed and potentially involved in related biological processes. A signed network approach was utilized, meaning that the directionality of gene correlations (positive or negative) was preserved to capture more nuanced biological relationships.

For the subset of 106 BD samples, the same WGCNA procedure was applied, but with a soft-thresholding power of 16 to accommodate the smaller sample size. This analysis aimed to identify gene modules specific to BD, uncovering potential molecular mechanisms that contribute to the age of onset in BD patients.

##### Module–Trait Relationship Analysis

To investigate the clinical relevance of the identified modules, the module eigengene (the first principal component representing the overall expression profile of a module) was calculated for each module [83,84]. Pearson’s correlation analysis was then performed between the module eigengenes and clinical traits, age of onset, and sex. Visualization and Interpretation Heatmaps of module–trait correlations allowed for the identification of modules strongly associated with age of onset and sex. These modules were further prioritized for downstream analyses by including Gene Ontology (GO) and Kyoto Encyclopedia of Genes and Genomes (KEGG) pathway enrichment analyses to understand the biological processes and pathways involved in the onset of BD and SCZ [85,86].

#### 4.2.2. Estimation of Bulk RNA-seq-Derived Relative Cell Type Proportions (rCTPs)

To estimate the rCTPs in our bulk RNA-seq datasets, we employed the mgpEstimate() function from the MarkerGeneProfile (MGP) R package v1.0.4.9000 (PavlidisLab; accessed on 26 November 2025), following methods described in previous studies [87]. Using a curated list of cell type-specific marker genes, we calculated the rCTPs for each sample and *z*-score normalized these estimates within each dataset before proceeding with downstream analyses.

To evaluate the robustness of our findings, we conducted two types of comparisons:

- Differential Gene Expression Analysis Results: We compared the significant genes identified from our DGE analysis with the MGP dataset to assess overlap between gene expression patterns and estimated cell type proportions. Fisher’s exact test was used to determine the statistical significance of the overlap.
- WGCNA Modules: We performed correlation analyses between module eigengenes (derived from WGCNA) and the rCTPs to identify modules that showed significant associations with specific cell types.

#### 4.2.3. Functional Enrichment Analysis

##### Gene Set Enrichment Analysis (GSEA)

GSEA was conducted using the clusterProfiler package v4.12.6 (Bioconductor; accessed on 26 November 2025) in R v4.4.1 [88,89,90]. GO and KEGG pathway enrichment analyses were performed on ranked gene lists from the differential expression analysis using clusterProfiler together with org.Hs.eg.db v3.19.1, DOSE v3.30.5, and enrichplot v1.24.4 (all Bioconductor; accessed on 26 November 2025). The input for GSEA consisted of *t*-values derived from the linear model results, ranked and mapped to Entrez IDs. The threshold for pathway significance was set at a corrected *p*-value < 0.25, following established guidelines for GSEA significance thresholds [89,91].

After WGCNA, for each module of interest that showed a significant correlation with age of onset (adjusted *p* < 0.05), GO and KEGG pathway enrichment analyses were conducted using the DAVID [60,61] and FUMA [92] platforms. In these analyses, genes from significant modules were analyzed to reveal their involvement in relevant biological processes, particularly focusing on calcium signaling pathways and related processes.

### 4.3. Machine Learning Model for Age of Onset Prediction

This study employed a two-stage machine learning framework to predict the likelihood of early-onset SCZ and BD (defined as onset before 18 years) using transcriptomic data from 369 individuals in the CMC dataset. For classification analyses, age of onset was dichotomized as early-onset (<18 years) versus late-onset (≥18 years). Although earlier literature often defines early onset as <21 years in BD and <18 years in SCZ, we adopted a unified threshold of <18 years to harmonize classification across diagnoses and prevent class imbalance between BD and SCZ groups [6,7,8]. This threshold falls within the widely accepted early-onset range in psychiatric genetics and was selected after confirming that results were qualitatively consistent when diagnosis-specific cutoffs (<21 BD/<18 SCZ) were applied in sensitivity analyses [6,7,8]. The objective of the machine learning analysis was to identify transcriptomic features that differentiate early-onset from late-onset cases and develop a robust classification model.

#### 4.3.1. Cross-Validation Strategy

To ensure robust estimation of model performance and prevent information leakage, we employed a nested cross-validation framework. The dataset was partitioned into five stratified folds. In each iteration of the outer loop, one-fold was held out as an independent test set, while the remaining four folds served as the tuning set for model development. Within each tuning set, hyperparameters were optimized using repeated 5-fold cross-validation.

The full nested cross-validation procedure (outer 5-fold CV × inner repeated 5-fold CV) was repeated 10 times using different random seeds to provide stable performance estimates. All reported results reflect aggregated performance across all outer test folds and repetitions.

#### 4.3.2. Two-Stage Machine Learning Pipeline

##### Stage 1: Transcriptomic Feature Selection Using LASSO Regression

To identify transcriptomic features associated with variation in age of onset, we applied LASSO regression to the continuous age-at-onset variable. LASSO’s sparsity-inducing penalty enables effective dimensionality reduction in high-dimensional gene expression data. Hyperparameters were tuned within the inner cross-validation loop, and only features with non-zero coefficients across folds were retained for downstream classification.

##### Stage 2: Classification of Early vs. Late Onset

The subset of genes selected in Stage 1 was used as input to a Logistic Regression classifier with L2 regularization to classify individuals as early-onset (<18 years) or late-onset (≥18 years). The Logistic Regression regularization parameter was optimized within the inner cross-validation loop to ensure that all tuning was independent of the held-out test samples in the outer loop.

#### 4.3.3. Evaluation Metrics

Model performance was evaluated exclusively on the independent outer test folds. For each outer fold, performance was quantified using the Area Under the Receiver Operating Characteristic Curve (AUC), and the mean AUC across all outer test folds and repetitions served as the primary evaluation metric.

## 5. Conclusions

Together, these findings support a three-component molecular framework for early-onset psychiatric illness: disruption of calcium channel and calcium-buffering systems; sex-specific modulation of cAMP signaling; and activation of stress-responsive MAPK pathways in bipolar disorder. This integrated model offers a biologically grounded explanation for how early-onset BD and SCZ may emerge during sensitive neurodevelopmental periods, particularly within prefrontal circuits. The patterns observed here align with broader evidence of cortical vulnerability, including alterations in structure, connectivity, and synaptic integrity, suggesting that transcriptomic dysregulation reflects circuit-level disruptions relevant to early illness onset.

Importantly, our machine-learning analyses converged with the transcriptomic and co-expression results: the top predictive features were strongly enriched for calcium-related signaling genes. This cross-modal convergence underscores the central role of calcium-mediated processes as a core molecular axis of early-onset vulnerability.

### Clinical Implications and Future Directions

The results of this study point to several clinically meaningful avenues. First, transcriptomic markers involved in calcium signaling pathway may contribute to earlier detection and risk stratification, particularly in adolescents showing emerging symptoms. Second, the female-specific involvement of the cAMP–CREB axis suggests opportunities for sex-sensitive risk monitoring and preventive strategies during adolescence.

Therapeutically, our findings point to several biologically tractable pathways that may inform future intervention strategies. Isoform-specific adenylate cyclases, which function within defined enzymatic and subcellular compartments, represent one potential avenue for targeted modulation. Similarly, pathways involving calcium signaling, cyclic nucleotide regulation, and MAPK-related stress responses provide additional conceptual entry points for exploring mechanism-based treatments. Although current agents acting on these systems are not used clinically for bipolar disorder or schizophrenia, they illustrate how the molecular signatures identified in this study may guide future therapeutic development and refinement. Overall, our integrative framework highlights interconnected transcriptomic and pathway-level alterations spanning calcium influx, intracellular buffering, cAMP signaling, and MAPK-related stress responses. These insights may support the development of more informative biomarkers, guide early-intervention strategies, and contribute to emerging precision-psychiatry approaches that account for sex-specific biological differences in early-onset bipolar disorder and schizophrenia.

## Figures and Tables

**Table 1:** Demographic data of CMC samples.

Characteristic	Male (*n* = 191)	Female (*n* = 178)	Overall (*n* = 369)
Diagnosis, *n* (%)			
Schizophrenia	148 (77.5%)	115 (64.6%)	263 (71.3%)
Bipolar Disorder	43 (22.5%)	63 (35.4%)	106 (28.7%)
Age of Onset (years)			
Mean ± SD	22.2 ± 8.5	22.0 ± 7.9	22.1 ± 8.2
Median (min-max)	-	-	21.0 (7.0–48.0)
Age of Death (years)			
Mean ± SD	46.2 ± 15.3	45.5 ± 14.7	45.9 ± 14.9
Median (min-max)	-	-	46.0 (17.0–83.4)
RIN			
Mean ± SD	7.5 ± 0.8	7.8 ± 0.7	7.7 ± 0.8
Median (min-max)	-	-	7.7 (5.1–9.4)
PMI (hours)			
Mean ± SD	38.6 ± 26.7	36.4 ± 24.1	37.4 ± 25.6
Median (min–max)	-	-	32.0 (5.5–168.0)
pH			
Mean ± SD	6.4 ± 0.4	6.4 ± 0.4	6.4 ± 0.4
Median (min-max)	-	-	6.4 (5.7–7.0)
White race, *n* (%)	142 (74.3%)	137 (76.9%)	279 (75.6%)

CMC: CommonMind Consortium; RIN: RNA Integrity Number; PMI: Post-Mortem Interval; NIMH_HBCC: National Institute of Mental Health Human Brain Collection Core; BrainGVEX: Brain Genomics Superstruct Project; LIBD_szControl: Lieber Institute for Brain Development Schizophrenia Control.

## Data Availability

The original contributions presented in this study are included in the article/Supplementary materials. Further inquiries can be directed to the corresponding authors.

## Supplementary Materials

The following supporting information can be downloaded at: https://www.mdpi.com/article/10.3390/ijms262412109/s1.
