# Supplementary figures and images for: Molecular Signatures of Early-Onset Bipolar Disorder and Schizophrenia: Transcriptomic and Machine-Learning Insights into Calcium and cAMP Signaling, Including Sex-Specific Patterns
Source: Int J Mol Sci. 2025 Dec 16;26(24):12109. doi: 10.3390/ijms262412109 (PMC12732934; doi:10.3390/ijms262412109)

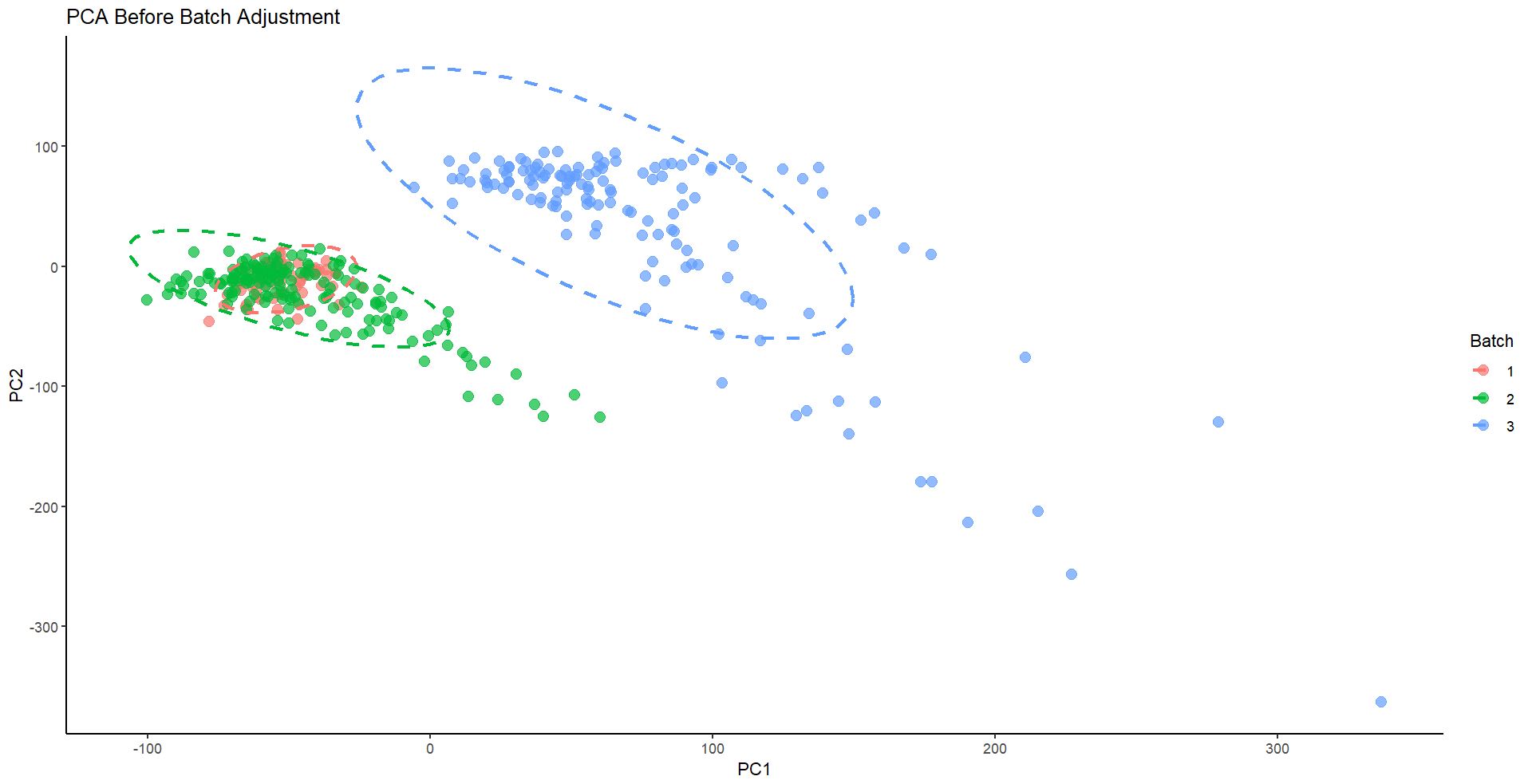

Supplement: Supplementary file 1 [file ijms-26-12109-s001.zip › FigureS1A.PCA_BEFORE PCA1vs2.JPG]

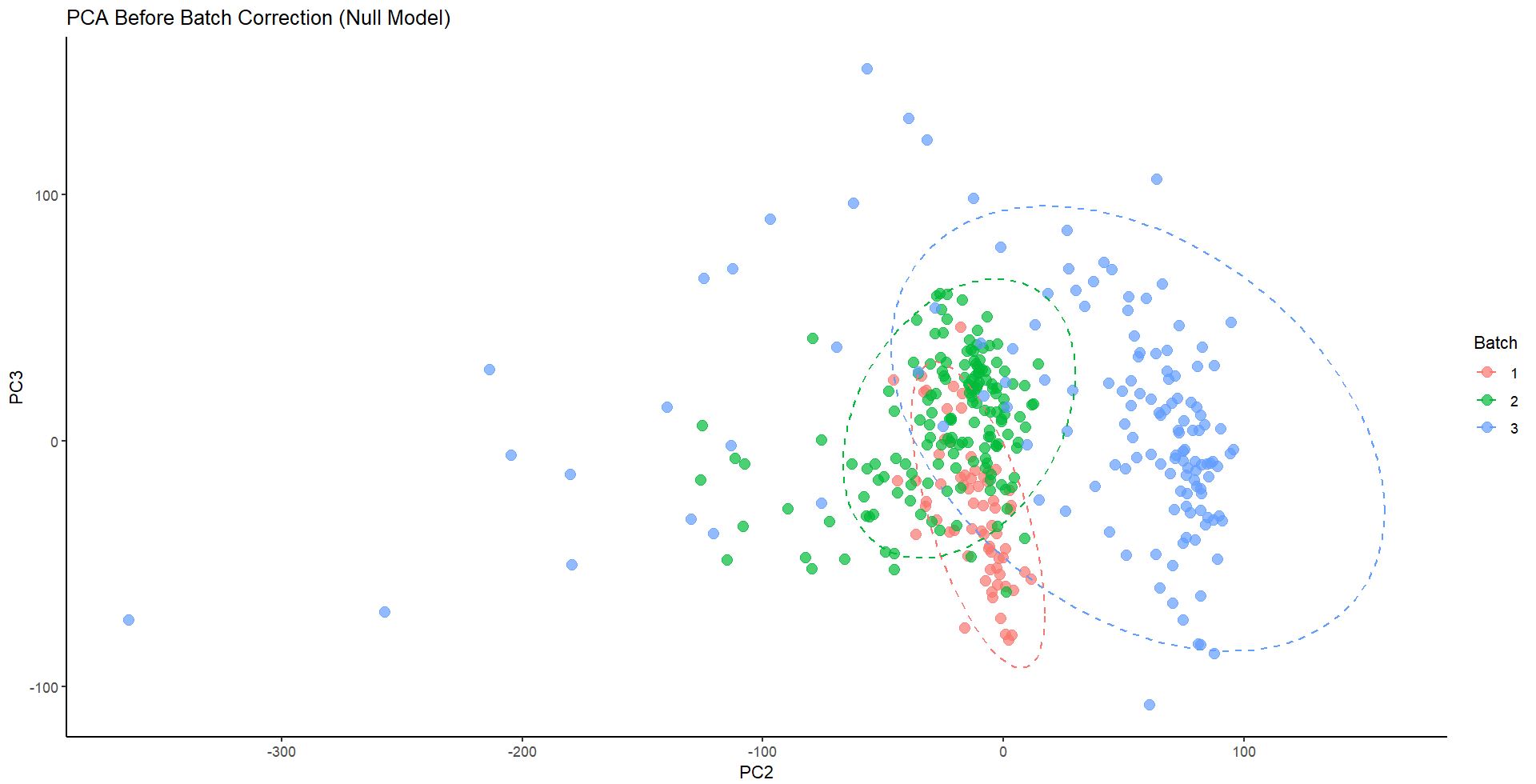

Supplement: Supplementary file 1 [file ijms-26-12109-s001.zip › FigureS1B.PCA_BEFORE PCA2vs3.JPG]

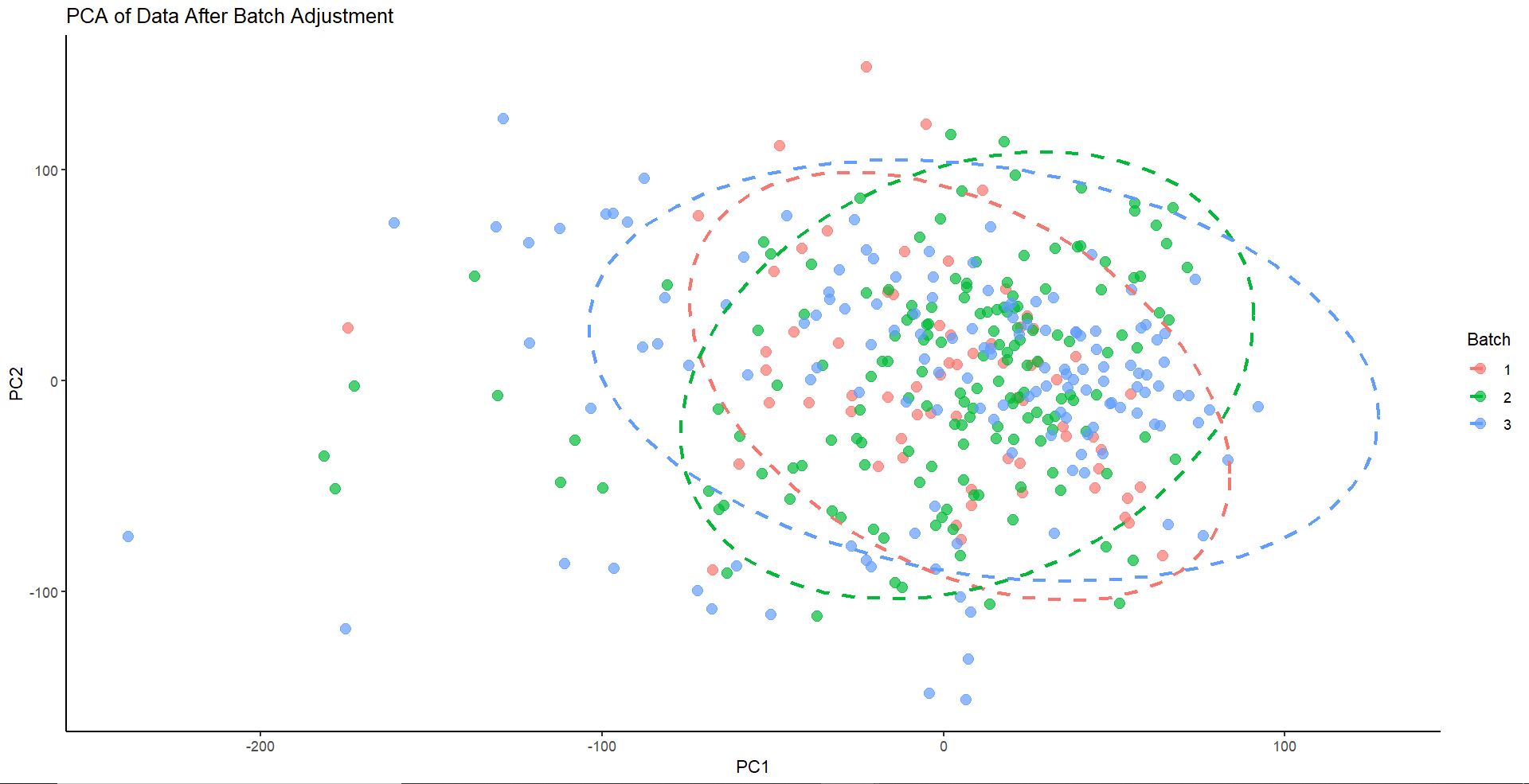

Supplement: Supplementary file 1 [file ijms-26-12109-s001.zip › FigureS1C.PCA_AFTER PCA1vs2 (1).JPG]

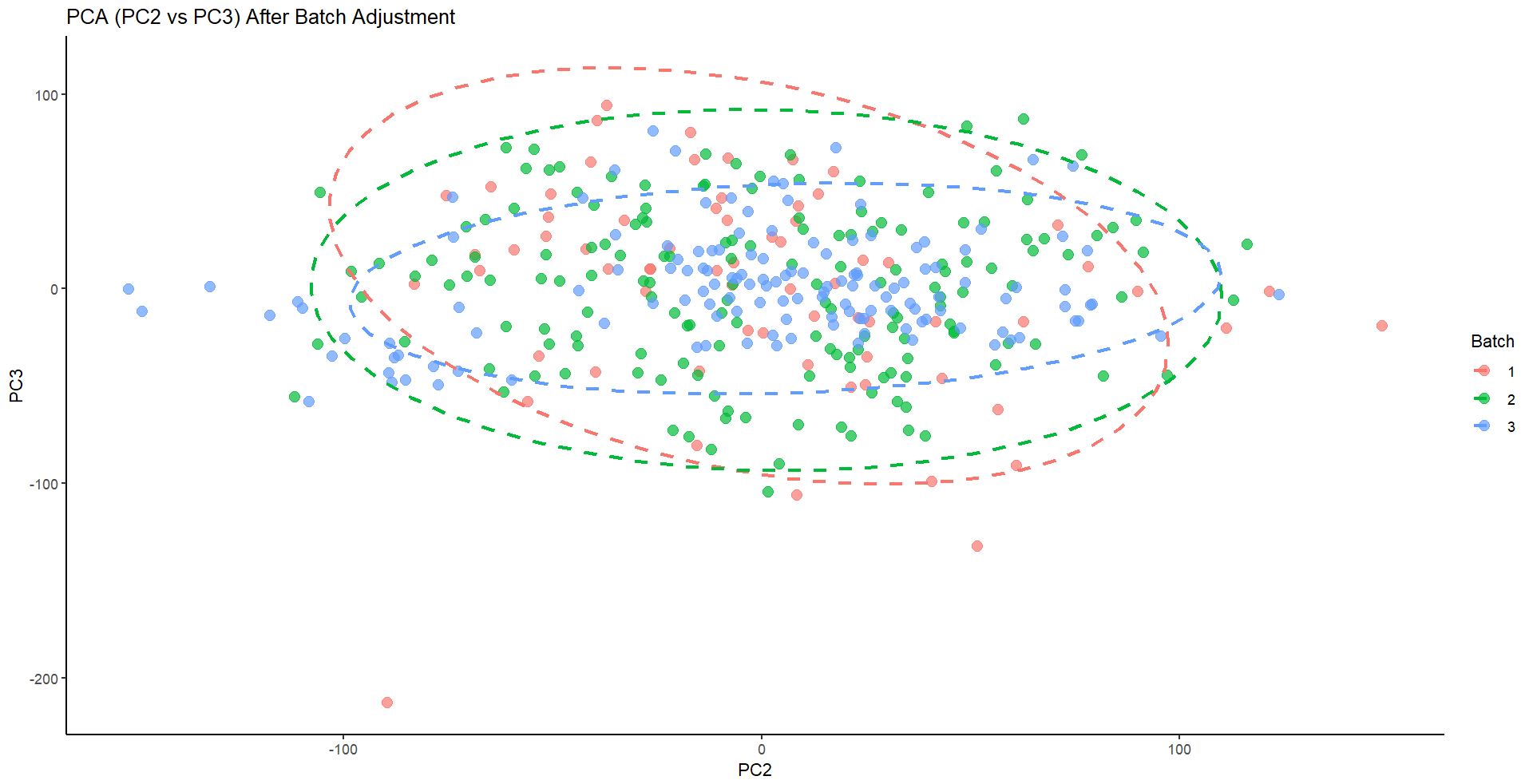

Supplement: Supplementary file 1 [file ijms-26-12109-s001.zip › FigureS1D.PCA_AFTER PCA2vs3 (2).JPG]

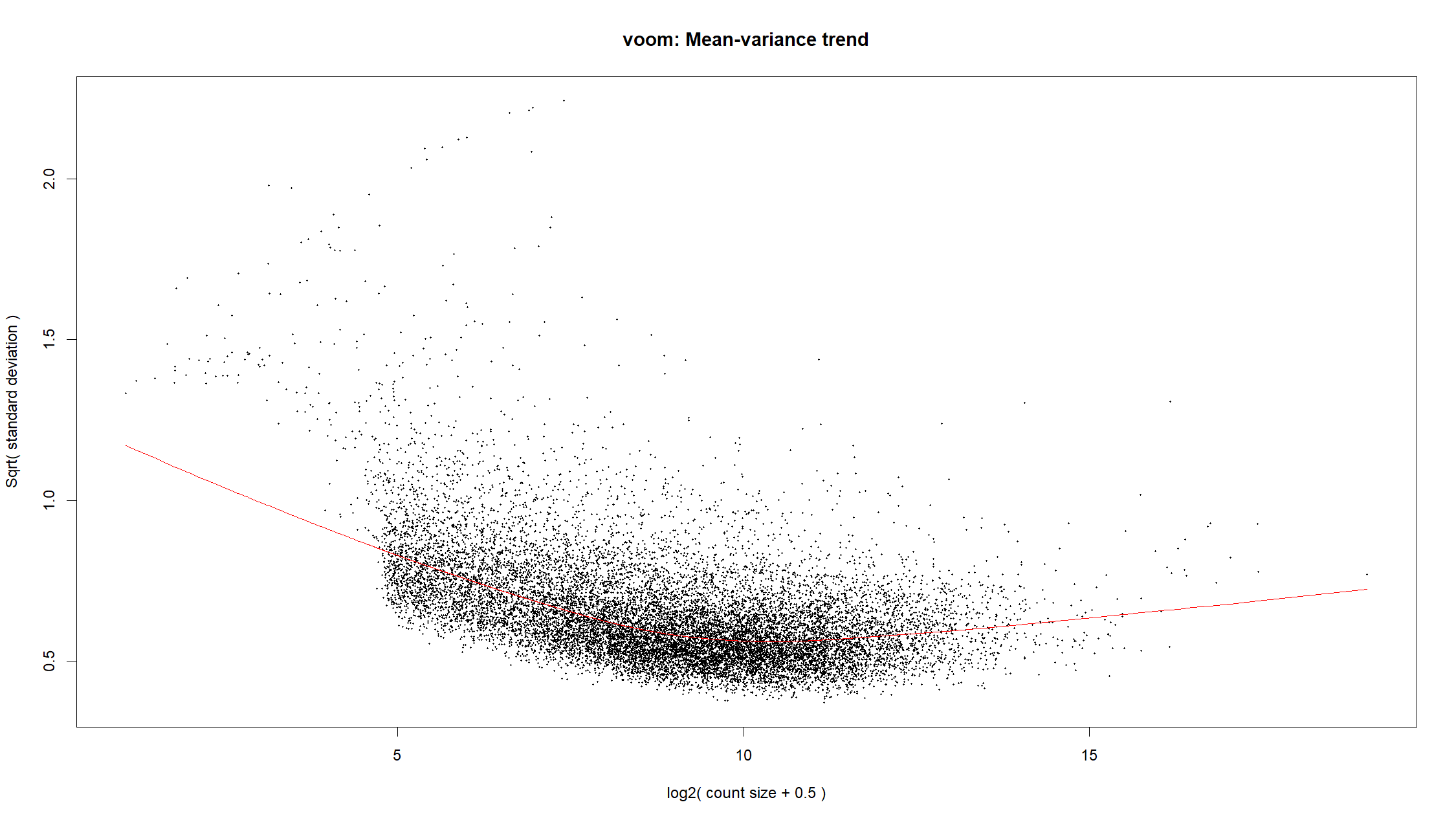

Supplement: Supplementary file 1 [file ijms-26-12109-s001.zip › FigureS1E.VOOM.png]

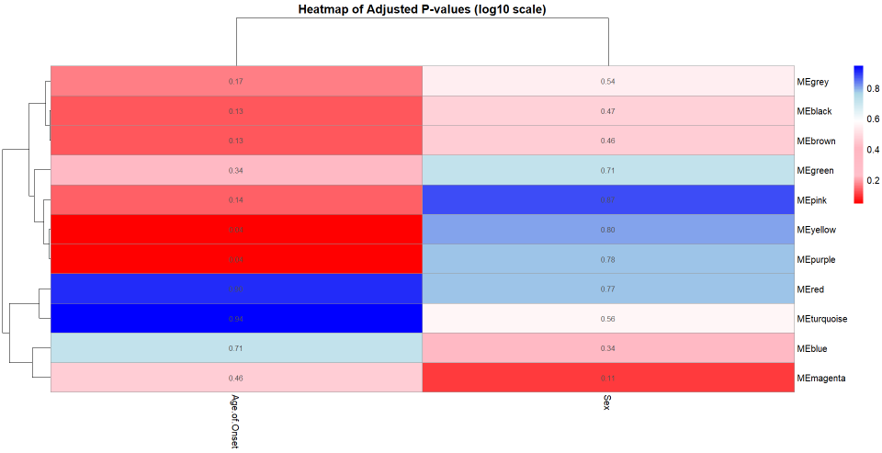

Supplement: Supplementary file 1 [file ijms-26-12109-s001.zip › FigureS2A.jpg.png]

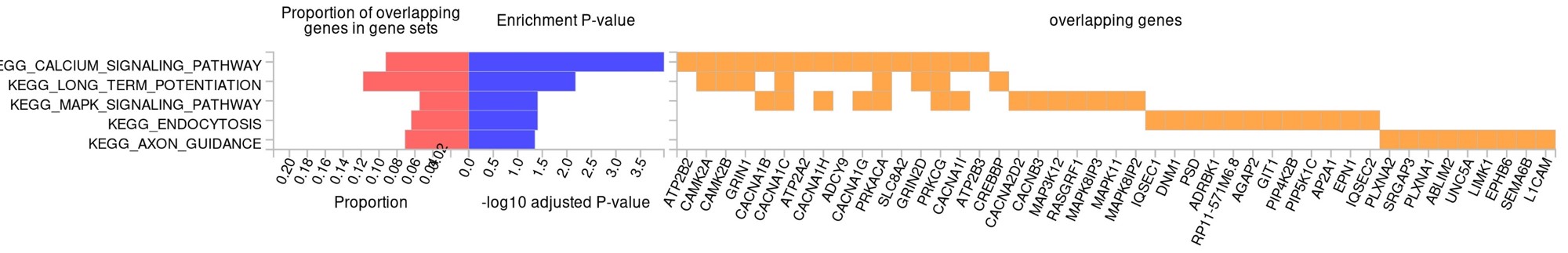

Supplement: Supplementary file 1 [file ijms-26-12109-s001.zip › FigureS2B.jpg]

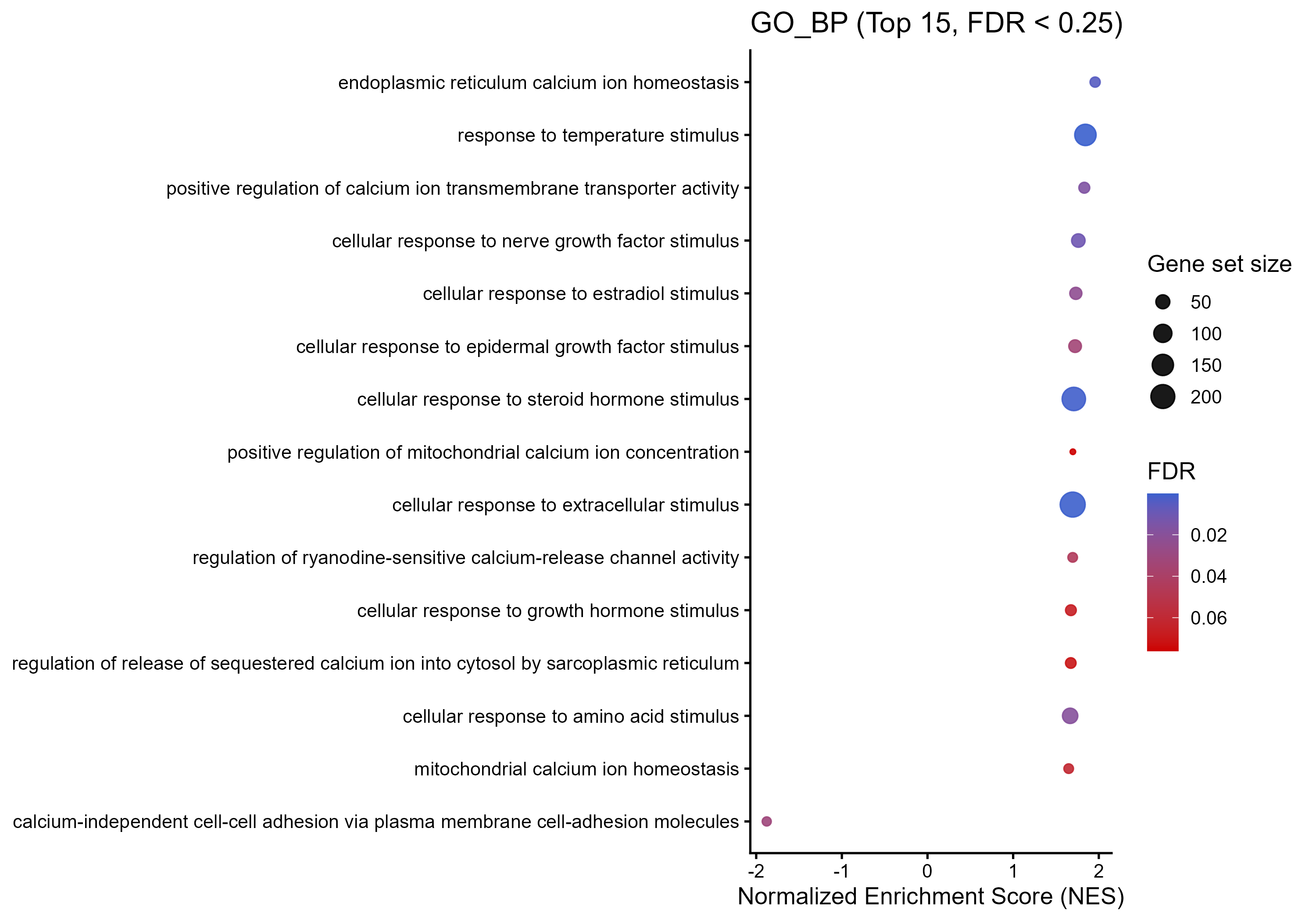

Supplement: Supplementary file 1 [file ijms-26-12109-s001.zip › FigureS3_Age_of_Onset_DGE_GO_BP_dotplot_Top15.png]

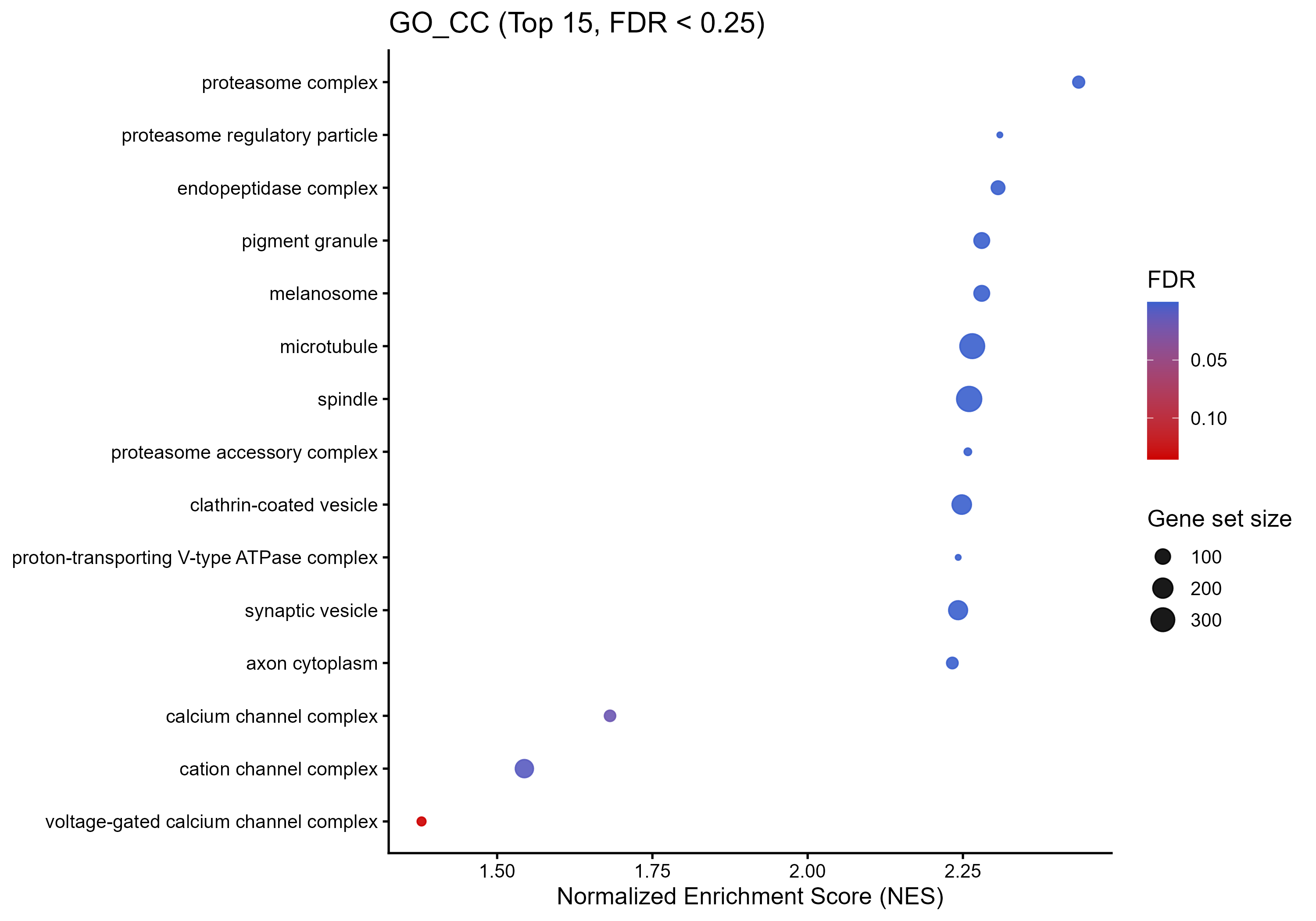

Supplement: Supplementary file 1 [file ijms-26-12109-s001.zip › FigureS4_Age_of_Onset_DGE_GO_CC_dotplot_Top15.png]

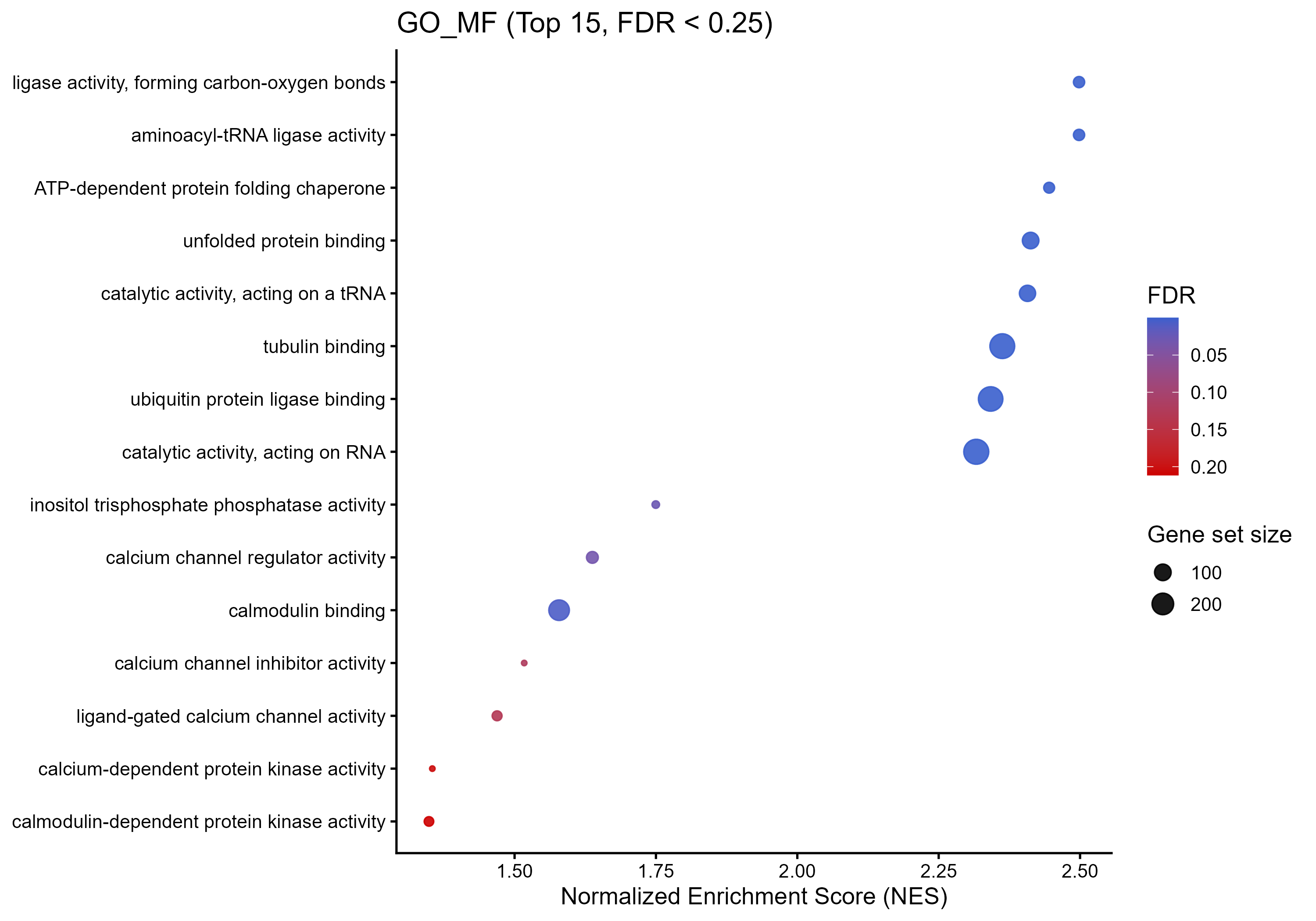

Supplement: Supplementary file 1 [file ijms-26-12109-s001.zip › FigureS5_Age_of_Onset_DGE_GO_MF_dotplot_Top15.png]

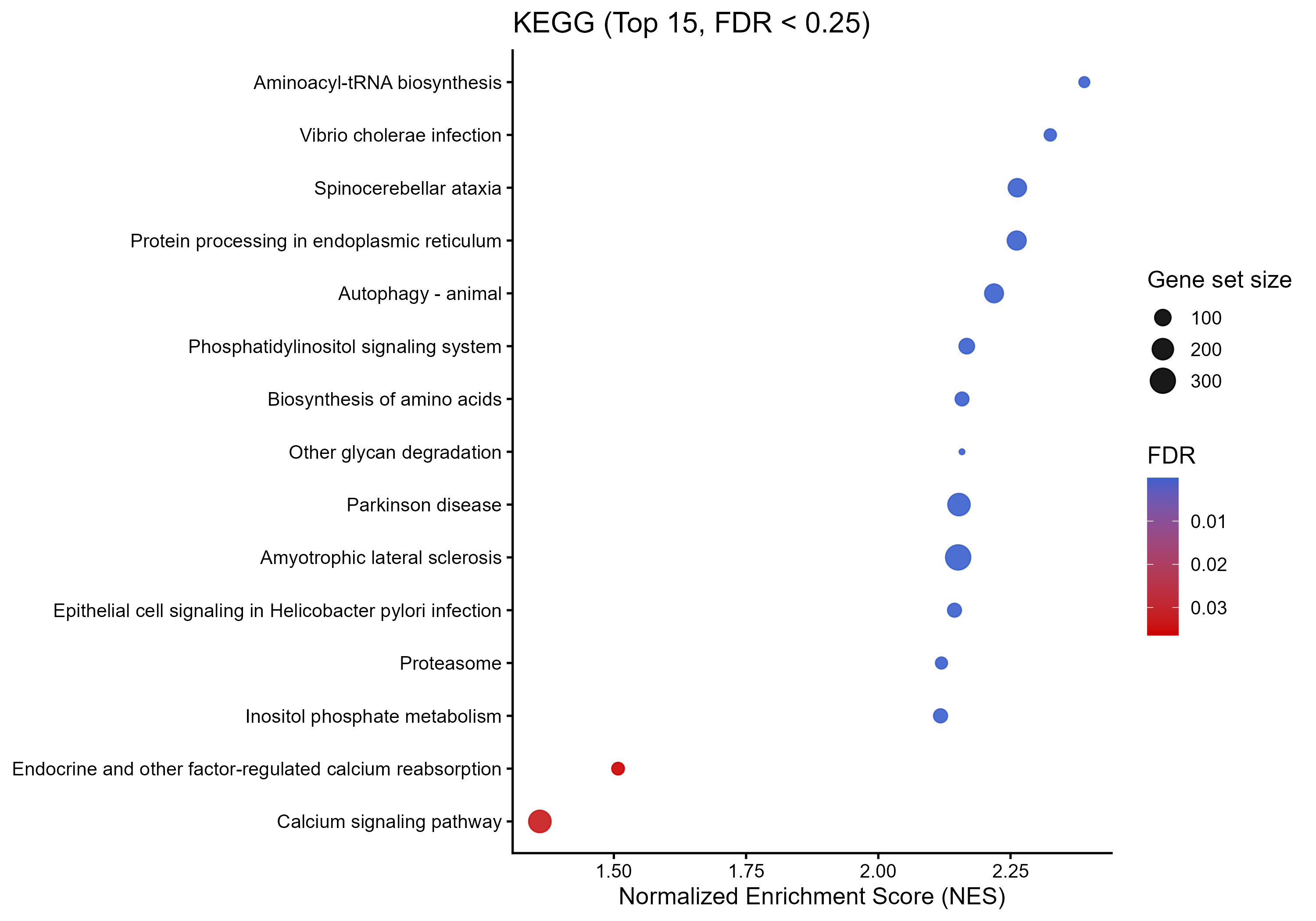

Supplement: Supplementary file 1 [file ijms-26-12109-s001.zip › FigureS6_Age_of_Onset_DGE_KEGG_dotplot_Top15.png]

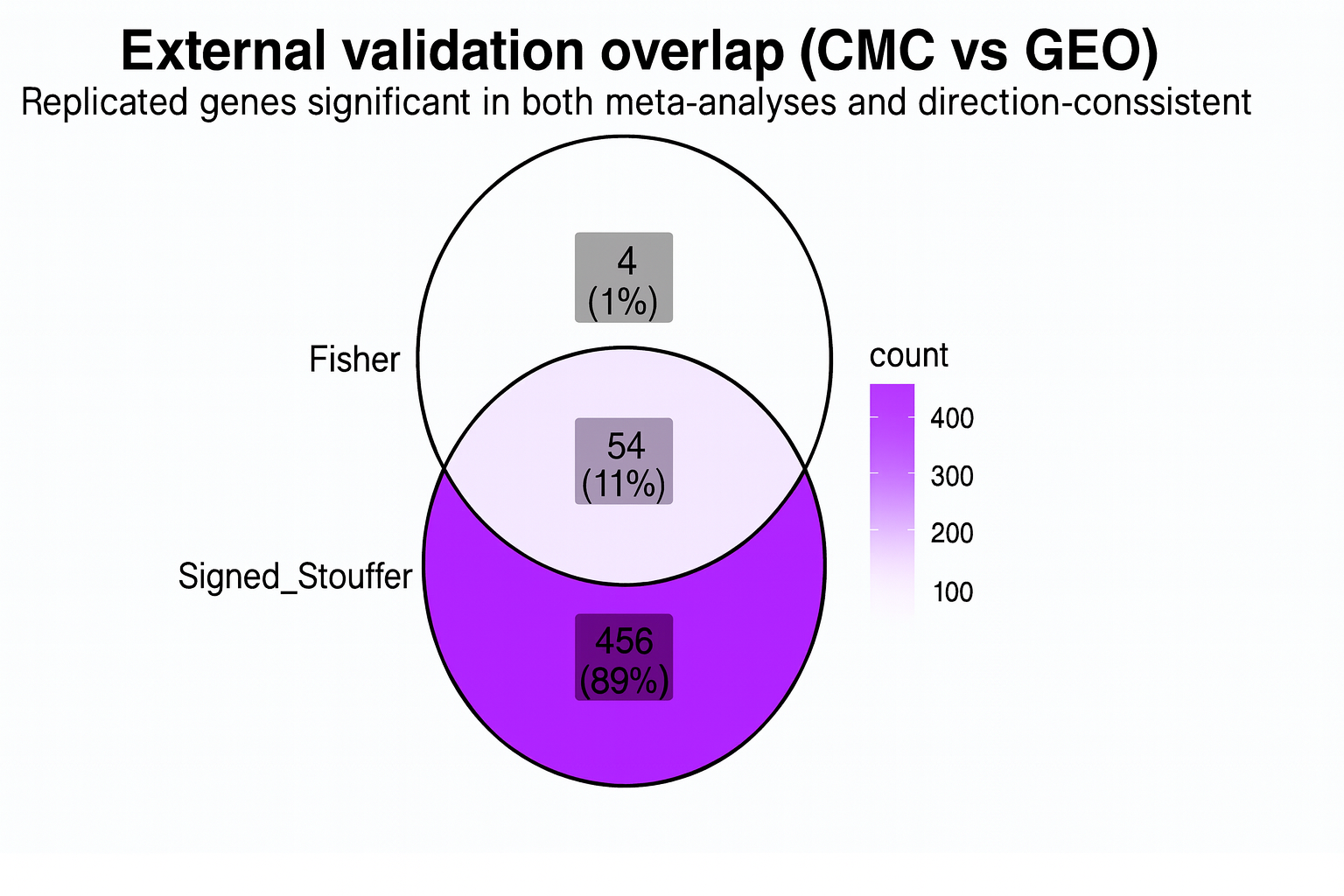

Supplement: Supplementary file 1 [file ijms-26-12109-s001.zip › FigureS7.Diagram.png]
